# A Rare Case of Pilomatricoma of the Male Breast Mimicking Breast Carcinoma: A Diagnostic Challenge

**DOI:** 10.7759/cureus.98654

**Published:** 2025-12-07

**Authors:** Elena Wen Xuan Tay, Naman Siddique, Olga Shmuilovich, Anat Kornecki, Mariamma G Joseph

**Affiliations:** 1 Department of Health Sciences, Western University, London, CAN; 2 Department of Medical Imaging, London Health Sciences Centre/Western University, London, CAN; 3 Department of Medical Imaging, St. Joseph's Health Centre/Western University, London, CAN; 4 Department of Pathology and Laboratory Medicine, London Health Sciences Centre/Western University, London, CAN

**Keywords:** benign skin adnexal tumors, beta-catenin ctnnb1, diagnostic challenges, male breast tumor, pathology, pilomatricoma, radiology

## Abstract

Pilomatricoma is a benign skin adnexal tumour often seen in children and young adults. We report a rare case of pilomatricoma of the breast in a 65-year-old male patient who presented with a palpable breast lump. Initial clinical and radiological evaluation suggested primary breast carcinoma. Histopathological examination showed classic features of a benign pilomatricoma with basaloid matrical cells, shadow (ghost) cells, and a foreign body giant cell reaction. There was only minimal microcalcification present. There was strong and diffuse nuclear and cytoplasmic staining of basaloid matrical cells for beta-catenin. This case highlights the diagnostic challenges posed by uncommon breast masses and emphasises the need for histologic confirmation by a tissue biopsy. Pathologists and clinicians should be aware that although pilomatricoma is a common skin adnexal tumour in the head and neck region, it can rarely present as a breast lump. Recognising this uncommon presentation is essential to avoid misdiagnosis and ensure appropriate management.

## Introduction

Pilomatricoma (PLM), also previously known as calcifying epithelioma of Malherbe, is a benign, slowly growing skin adnexal tumour showing hair follicle differentiation towards the matrical portion of the hair bulb and hair cortex [1]. It occurs commonly in children and young adults as a solitary papule or nodule, mostly in the head and neck region (50%), followed by extremities (upper 25%, lower 10%) and trunk (15%) [1]. This neoplasm usually presents as a firm and painless dermal or subcutaneous mass. PLM of the breast is exceptionally rare, and the diagnostic challenge with this tumour lies in its ability to mimic more common aggressive malignant breast lumps, both in clinical presentation and imaging findings [2]. Here, we describe the clinicopathological and imaging findings of PLM of the breast in a male patient and highlight the importance of including this tumour in the differential diagnoses of patients presenting with breast lumps.

## Case presentation

A 65-year-old man presented with a slightly painful palpable lump on his left breast. He first noticed the lesion six months ago and has observed only slow growth since then. On physical examination, he had a multinodular firm mass measuring 2-3 cm in diameter, located on the upper outer quadrant of the left breast. The mass was slightly tender on palpation and firm but not fixed to the underlying fascia. There was slight erythema and stretching of the overlying skin, but no ulceration. There was no associated cervical, supraclavicular, infraclavicular or axillary lymphadenopathy. The patient was evaluated at the Breast Imaging Centre, and a clinical/imaging suspicion of primary breast carcinoma was raised. He underwent prompt surgical excision of the mass with adequate margins for diagnostic purposes. A prior ultrasound-guided core biopsy was not performed for this patient.

Radiology findings

**Figure 1:**
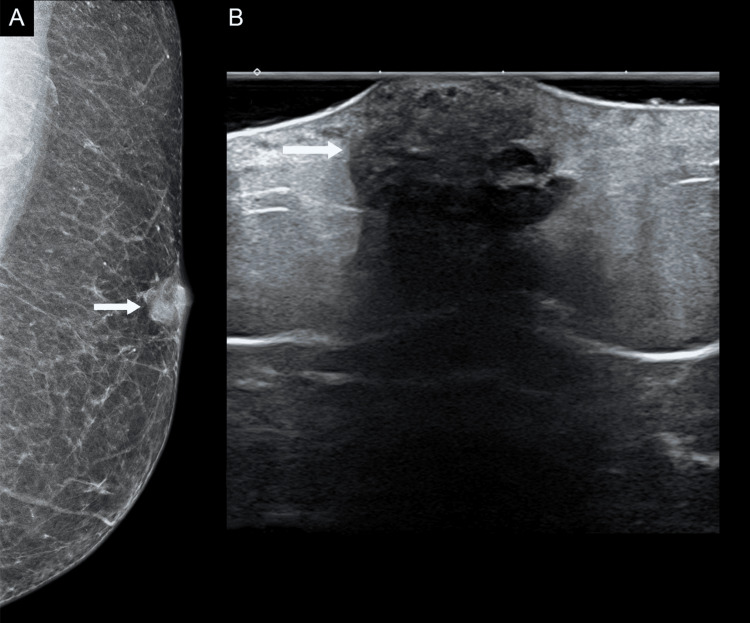
Radiologic Findings of PLM 1A: The mammography image demonstrates a well-circumscribed, irregular, dense mass in the upper outer quadrant of the left breast measuring 1.5 x 1.2 cm with no associated microcalcifications. 1B: The ultrasound image shows an irregular, heterogeneously hypoechogenic solid mass in the upper outer quadrant of the left breast measuring 1.6 x 1.7 cm.

Diagnostic mammography showed a well-circumscribed, irregular dense mass in the upper outer quadrant of the left breast measuring 1.5 x 1.2 cm with no associated microcalcifications. There was no associated lymphadenopathy. Subsequent breast ultrasound examination demonstrated an irregular, heterogeneously hypoechogenic solid mass with dense posterior shadowing and tiny cystic degeneration in the upper outer quadrant, measuring 1.6 x 1.7 cm, within the subcutaneous fat and invading the skin (Figures 1A, 1B).

Internal vascular flow was observed on colour Doppler. The radiology impression was suspicious for primary breast malignancy, categorised as BI-RADS 5.

Pathology findings

**Figure 2:**
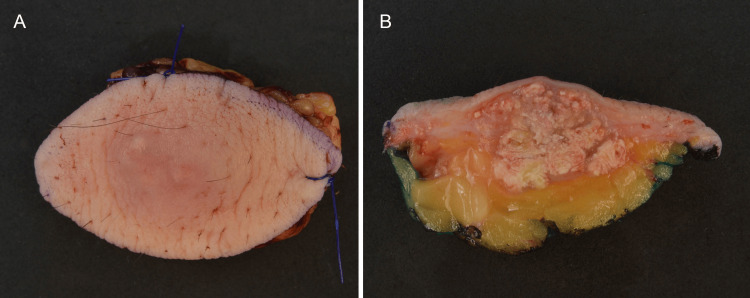
Gross Pathology of PLM Grossly, the resected specimen shows slightly erythematous stretched skin surface (2A) and an underlying tumour with a well-circumscribed lobulated tan-white cut surface (2B).

Gross examination of the excised breast specimen revealed a well-circumscribed, irregular, multinodular mass with dermal and subcutaneous involvement, measuring 1.7 x 1.3 x 1.2 cm. The cut surface was whitish, firm, and slightly lobulated (Figures 2A, 2B).

**Figure 3:**
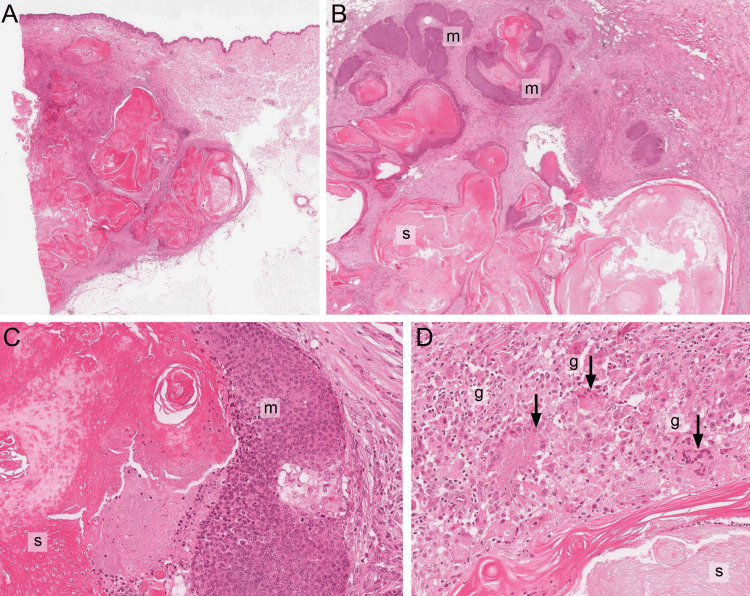
Microscopic Pathology of PLM Histologic examination of PLM (3A-3D) shows a well-circumscribed solid/cystic tumour composed of basaloid matrical cells (m), shadow cells (s) and a foreign body giant cell reaction (g, arrows) (haematoxylin and eosin stain, 2, 4, 10× magnification).

Histologic examination of the tumour showed a well-circumscribed dermal/subcutis-based benign tumour with classic features of PLM (Figures 3A-3D).

**Figure 4:**
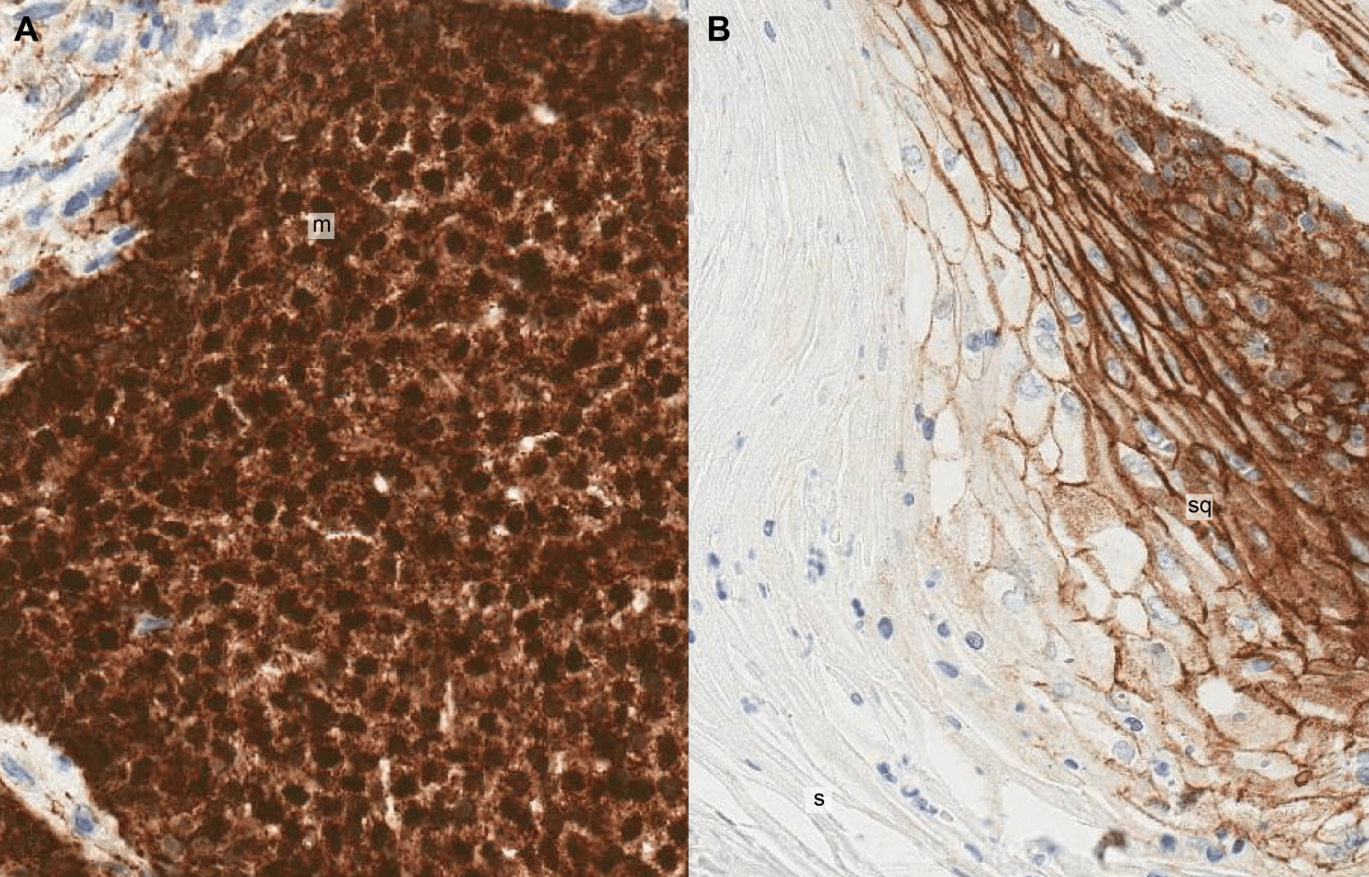
Immunohistochemistry of PLM 4A: There is strong and diffuse nuclear and cytoplasmic staining of basaloid matrical (m) cells for β-catenin (20× magnification). 4B: There is only membrane staining of nucleated squamous (sq) cells for β-catenin. Anucleate shadow cells (s) are negative for β-catenin (10× magnification).

This tumour was composed of multiple foci of basophilic matrical cells showing abrupt keratinisation with cystic change, abundant shadow (ghost) cells and marked foreign-body-type granulomatous inflammation. These mitotically active, benign matrical cells were oval-shaped with a high nuclear-cytoplasmic ratio and small nucleoli. Although mitotic figures were seen, there was no atypia or malignancy in these matrical cells. The tumour was completely excised with surgical margins clear of tumour. Immunohistochemistry demonstrated positive nuclear and cytoplasmic staining of the matrical cells for beta-catenin (Figures 4A, 4B). Given the diagnosis of benign PLM with clear surgical margins, no further therapy was given. Clinical follow-up was unremarkable with complete healing of the incision site.

## Discussion

Pilomatricoma (PLM), previously known as Calcifying Epithelioma of Malherbe, was initially described in 1880 by Dr. Chenantais Malherbe. This benign follicular adnexal tumour shows differentiation towards hair bulb matrical cells and hair cortex. This tumour is commonly seen in children and young adults, although a wide age range has been described, from 3 months to 93 years. Most studies have noted a slight female predominance. Usually, PLM presents as a solitary lesion, but approximately 5% of patients have multiple PLMs, usually in association with complex genetic syndromes such as myotonic dystrophy, Gardner syndrome, Turner syndrome, Rubinstein-Taybi syndrome, Goldenhar syndrome, Kabuki syndrome, Sotos syndrome, trisomy 9, gliomatosis cerebri, panhypopituitarism, MYH-associated polyposis and DICER1 syndrome [1].

Grossly, PLM usually appears as a circumscribed, firm to hard, white nodulocystic tumour with cheesy material. Microscopy shows a combination of basophilic matrical cells (which express nuclear and cytoplasmic β-catenin), shadow (ghost) cells, amorphous keratinous debris and variable granulomatous inflammation induced by keratin. The proportion of matricial cells to shadow cells decreases with increasing age of the lesion. Calcification and even ossification can occur in long-standing tumours. Rare histologic variants of PLM have been reported, such as perforating PLM, bullous PLM and pigmented PLM [1,2]. A rare presentation of PLM as a fungating breast mass was reported by Esponsito [3]. Most cases behave indolently without recurrences, and surgical excision with clear margins is the main mode of treatment with a low recurrence rate. Malignant transformation is exceedingly rare, requiring features that demonstrate infiltrating borders, solid aggregates of mitotically active malignant matrical cells, areas of necrosis and minimal shadow cells [1].

Our 65-year-old male patient presented with a palpable tumour in the upper outer quadrant of the left breast with a histologic diagnosis of PLM. In our literature search, only 13 cases of PLM of the breast have been reported, with 9 of these occurring in male patients [2-19]. The age of these 13 cases ranged from 13 to 69 years, with an M:F ratio of 9:4. The age of nine male patients ranged from 36 to 69 years, and all presented with palpable firm breast masses. Most PLMs were associated with microcalcifications on imaging, and all patients were treated with surgical excision. In the previous reports, these tumours have been documented in the central peri-areolar region as well as in all quadrants of the breast. On ultrasound imaging, they were often assigned a BI-RADS 4-5 score, reflecting a suspicious abnormality that warranted tissue diagnosis. These cases emphasise the rarity and importance of considering PLM in the differential diagnosis of breast masses in both male and female patients. Since the clinical and imaging findings are somewhat similar in breast carcinoma and PLM, histologic evaluation (core biopsy or excisional biopsy) should be performed to confirm the diagnosis, and surgical excision with clear margins would be the standard of treatment.

Pathogenesis of PLM is recently thought to be associated with mutations in exon 3 of the gene (CTNNB1) encoding beta-catenin; this 92 kDa cytoplasmic protein encoded by the gene CTNNB1 is involved in cell-cell adhesion as well as in the WNT signalling pathway, which regulates cell proliferation, differentiation and survival [1]. In addition, the matrical cells often harbour trisomy of chromosome 18, which carries the apoptotic BCL2 gene, suggesting that the BCL2 oncoprotein may also play a role in the growth and differentiation of PLM [17]. β-catenin expression in normal hair matrical cells, cortex cells and variably in outer root sheath cells of normal hair follicles also signifies its importance in hair follicle development and thus the origin of PLM from hair follicles [17]. In our case of PLM, there was variable expression of β-catenin across different cellular components of this tumour, consistent with the diagnosis. There was strong and diffuse β-catenin nuclear and cytoplasmic staining in basaloid matrical cells (Figure 4A). The transitional mature nucleated squamous cells showed only membrane β-catenin staining along the intercellular junctions as seen in normal epidermal keratinocytes. The shadow (ghost) cells were negative for β-catenin expression (Figure 4B).

## Conclusions

Our case report is unique because it involves an elderly male patient who presented with a palpable, slightly painful, firm breast lump on his left breast that appeared suspicious for breast carcinoma on imaging (BI-RADS 5). However, this breast mass was ultimately diagnosed by histopathology as pilomatricoma, a benign skin adnexal tumour showing follicular differentiation. Recognising this exceedingly rare presentation of this tumour as a breast lump is essential to avoid misdiagnosis and ensure appropriate surgical management.

This case underscores the diagnostic challenge posed by pilomatricoma, a benign tumour, in the evaluation of breast lumps. In addition, using immunohistochemical stains, we have contributed to the expanding understanding of the role of β-catenin in the pathogenesis of pilomatricoma.

## References

[1] WHO Classification of Tumours Editorial Board (2025). Skin Tumours. https://publications.iarc.who.int/Book-And-Report-Series/Who-Classification-Of-Tumours/Skin-Tumours-2025.

[2] Jones CD, Ho W, Robertson BF, Gunn E, Morley S (2018). Pilomatrixoma: a comprehensive review of the literature. Am J Dermatopathol.

[3] Esposito SB, Perez CB (2024). Rare presentation of pilomatrixoma as a fungating breast Mass: case report and literature review. Surg Case Rep Surg.

[4] Al-Issai M, Al-Rahbi S, Al-Futaisi A (2023). Pilomatrixoma of the male breast: case report and literature review. Oman Med J.

[5] AlSharif S, Meguerditchian A, Omeroglu A (2015). Pilomatricoma of the male breast: sonographic mammographic MRI features with pathologic correlation. Clin Imaging.

[6] Gil JR, Herh SJ, Kim Y (2016). A pilomatricoma misdiagnosed as male breast cancer. J Korean Soc Radiol.

[7] Ward RC, Donegan L, Khalil H, Wang Y (2019). Pilomatricoma of the male breast. Breast J.

[8] Kapoor A, Narayanan R, Tandon A, Santosh AK (2018). Pilomatricoma: an unusual cause of lump in a male breast. J Clin Ultrasound.

[9] Clark A, Leddy R, Spruill L, Cluver A (2019). Pilomatrixoma, a rare mimicker of male breast cancer. J Clin Imaging Sci.

[10] Ismail W, Pain S, Al‐Okati D, Sewan M. (2000). Giant pilomatricoma simulating carcinoma of the male breast. Int J Clin Pract.

[11] Becker TS, Moreira MAR, Lima LA (2010). Pilomatrixoma mimicking breast cancer in man. Breast J.

[12] Gilles R, Guinebretière JM, Gallay X, Vanel D (1993). Pilomatrixoma mimicking male breast carcinoma on mammography. AJR Am J Roentgenol.

[13] Hamilton A, Young GI, Davis RI (1987). Pilomatrixoma mimicking breast carcinoma. Br J Dermatol.

[14] Sood N, Raj B (2021). Pilomatricoma male breast, mimicking breast carcinoma-A rare case. Indian J Pathol Microbiol.

[15] Kim SJ (2013). Pilomatricoma of the breast in an adolescent girl: sonographic findings. J Med Ultrason.

[16] Hubeny CM, Sykes JB, O'Connell A, Dogra VS (2011). Pilomatrixoma of the adult male breast: a rare tumor with typical ultrasound features. J Clin Imaging Sci.

[17] Kim YS, Shin DH, Choi JS, Kim KH (2010). The immunohistochemical patterns of the β-catenin expression in pilomatricoma. Ann Dermatol.

[18] Taghipour Zahira S, Derakhshani F, Rahmani K (202411). Breast pilomatrixoma in a 47-year-old woman: pathological and radiological diagnosis. Arch Breast Cancer.

[19] Bensalah A, Benaaddach HO, Gouzi I (2021). Pilomatrixoma mimicking a breast neoplasm: imaging finding in an uncommon case report. Radiol Case Rep.

